# The Anti-allergic Cromones: Past, Present, and Future

**DOI:** 10.3389/fphar.2017.00827

**Published:** 2017-11-14

**Authors:** Ajantha Sinniah, Samia Yazid, Roderick J. Flower

**Affiliations:** ^1^Department of Pharmacology, Faculty of Medicine, University of Malaya, Kuala Lumpur, Malaysia; ^2^Trio Medicines Ltd., Hammersmith Medicines Research, London, United Kingdom; ^3^Centre for Biochemical Pharmacology, William Harvey Research Institute, St Barts and the Royal London School of Medicine, Queen Mary University of London, London, United Kingdom

**Keywords:** cromones, PKC, Annexin A1/FPR, GPR35, PP2A

## Abstract

The anti-allergic cromones were originally synthesized in the 1960s by Fisons Plc, and the first drug to emerge from this program, disodium cromoglycate was subsequently marketed for the treatment of asthma and other allergic conditions. Whilst early studies demonstrated that the ability of the cromones to prevent allergic reactions was due to their ‘mast cell stabilizing’ properties, the exact pharmacological mechanism by which this occurred, remained a mystery. Here, we briefly review the history of these drugs, recount some aspects of their pharmacology, and discuss two new explanations for their unique actions. We further suggest how these findings could be used to predict further uses for the cromones.

## History of Cromones

The prototypical cromone, *disodium cromoglycate*, (cromolyn sodium) was introduced into clinical medicine in the early 1960s by the pharmaceutical company Fisons Plc. It arose from a study of the anti-spasmodic properties of the Egyptian medicinal herb Khellin. The experimental trail leading to the discovery of the anti-allergic properties of this compound and its clinical validation by the Fisons’ pharmacologist, Roger Altounyan (himself an asthmatic), which entailed considerable self-experimentation, have become the stuff of pharmacological legend and will not be recounted here (see Howell, 2005). Originally, disodium cromoglycate was introduced for the treatment of mild-moderate allergic asthma. Although largely superseded by other medicines, it remains in the allergist’s cache today and has maintained a stature as an effective drug with a good safety margin. In 1970s, Fison’s developed nedocromil (Cairns et al., 1985), which not only shares a close chemical resemblance to cromoglycate, but also portrays comparable or even higher efficacy in the clinical setting. Subsequently, these drugs (often generically referred to as ‘cromones’) were also used to treat other allergic conditions (e.g., intestinal allergies, mastocytosis, and other allergic skin conditions).

Detailed research into the pharmacological actions of cromoglycate and nedocromil indicated that they inhibited mast cell degranulation provoked by various stimuli and thus these drugs were dubbed ‘mast cell stabilizers’ (Cox, 1967). In this respect, it was clear that the cromones had a distinctive mechanism of pharmacological action. Their ‘mast cell stabilizing’ effect (Vane, 1971; Thomson and Evans, 1973; Theoharides et al., 2000; Bandeira-Melo et al., 2005; Yazid et al., 2009) was clearly dissimilar to other drugs such as the β-agonists which, although more efficient at inhibiting mast cell degranulation (Shichijo et al., 1998), did not share other pharmacological characteristics of the cromones.

There are several disadvantages to using disodium cromoglycate therapeutically. It had to be administered at frequent intervals because of its poor pharmacokinetics and short half-life. Prophylactic treatment was essential and, for asthmatics, inhalation was the most reliable route of administration as this maximized the concentration in the lung. Despite this, and whilst inferior to the glucocorticoids for the treatment of asthma and allergies, these drugs have retained a niche role with a reputation for being very well tolerated and especially useful in pediatric formulations.

## Cromone Pharmacology

In addition to their effect on mast cells, several other actions of cromoglycate-like drugs have been reported which might be classified as ‘anti-inflammatory’. These include inhibition of activation (Kay et al., 1987), migration (Bruijnzeel et al., 1993; Szkudlińska et al., 1996) of polymorphonuclear (PMN) leukocytes; macrophage activation (Joseph and Rainey, 1992), mediator (Dahlén et al., 1989), and tachykinin action (Yamawaki et al., 1997); eicosanoid (Mattoli et al., 1990; Radeau et al., 1993) and cytokine release (Rusznak et al., 1996) as well as adhesion molecule expression (Hoshino and Nakamura, 1997; Okada et al., 1999; Sacco et al., 1999; Vural et al., 2000) and blockade of chloride channels (Alton and Norris, 1996; Heinke et al., 1995; Norris and Alton, 1996). It is not clear whether their mast cell stabilizing effect alone is responsible for their anti-asthmatic action in humans, (but it probably underlies their anti-allergic action), however, there is a widely-accepted notion that cromoglycate and nedocromil render their anti-asthmatic effects due to a combination of these anti-inflammatory actions (Cairns et al., 1985). Given that inflammatory diseases are associated with pro-inflammatory stimuli in pathological setting, ‘mast cell stabilizing’ properties of cromones were tested not only in the presence of Ig-E (Leung et al., 1988; Oka et al., 2012; Yazid et al., 2013), but also using the mast cell secretagogue such as compound 48/80 (Jeong et al., 2006; Sinniah et al., 2016). Nonetheless, typical inducers of inflammation such as lipopolysaccharide (LPS) (Nava and Caputi, 1999; Oka et al., 2012; Zhang X. et al., 2016; Hughes et al., 2017), phorbol 12-myristate 13-acetate (PMA) (Sadeghi-Hashjin et al., 2002; Yazid et al., 2009) and TNF-α (Bissonnette et al., 1995) were also utilized to elucidate the mechanism of action of cromone. To identify the pharmacological actions of cromones *in vivo* settings, various inflammatory models were used to assess the mast cell function (Wyss et al., 2005; Kneilling et al., 2007; Hei et al., 2008; Liu et al., 2009; Ramos et al., 2010; Zhang S. et al., 2016; Hughes et al., 2017).

Despite earlier studies led to the notion that the cromoglycate-like drugs acted largely on mast cells (Cox, 1967, 1970; Cox and Altounyan, 1970; Cox et al., 1970; Orr, 1989) to inhibit the release of mediators, current evidence suggest that this is unlikely to be their sole target of action and that these drugs do exert pharmacological effect in non-allergic settings. Indeed, work from our own group (Yazid et al., 2010b) have shown that cromones inhibit neutrophil recruitment onto vascular endothelium, further suggesting that these drugs could play a role in diseases such as gouty arthritis and vasculitis, which are steered by excessive PMN activation. Clearly, it could be argued that cromones impede PMN trafficking by inhibiting the release of mediators from mast cells, however, a study by Furuta et al. (1998), have shown that there is a separate and direct effect of cromones on PMN, which does not require mast cell participation. Interestingly, cromoglycate-like drugs also targets inhibition of eicosanoids release (Mattoli et al., 1990; Radeau et al., 1993; Yazid et al., 2009) and cytokine production (Kimata et al., 1994; Devalia et al., 1996; Yazid et al., 2013), which further reiterates the concept that cromones’ actions are not only limited to allergic inflammation.

## Potential Therapeutic Mechanisms

Early investigations of cromoglycate action on mast cell degranulation highlighted several unusual features. The cromones appeared to exhibit strong tachyphylaxis (Sung et al., 1977a,b; Church and Hiroi, 1987) and the timing of the drug administration relative to the degranulating agent was also crucial in determining their pharmacological effect (Shichijo et al., 1998). Tachyphylaxis is so prominent that the blocking effect of the drug often cannot be repeated within a defined time-frame (Thomson and Evans, 1973) and if the drug was administered too far in advance of the degranulating stimulus, it was ineffective. These observations have led to speculations that cromoglycate released a substance, which once exhausted, required replenishment before the next challenge could be effective (Thomson and Evans, 1973). Other incongruities observed with these drugs were connected with the optimal concentration required for the inhibition of mast cell degranulation *in vitro*, since there were discrepancies amongst species and between mast cell subtypes, in their response to cromones (Church and Hiroi, 1987; Kay et al., 1987; Pearce et al., 1982). One group has even questioned whether cromoglycate, which is highly active in the rat, has any efficacy in the mouse (Oka et al., 2012). An interesting observation has shown that cromoglycate induced the phosphorylation of a 78kD mast cell protein, which inhibits mast cell activation (Wells and Mann, 1983). However, in the presence of phorbol esters, these drugs loses this ability, and render inactive (Cox et al., 1998). This phenomenon further suggests that cromoglycate is involved in an endogenous control mechanism that switches off the release of mediators.

### A Role for PKC?

There have been dispersed reports of an association between cromoglycate and PKC over the past 3 decades (Lucas and Shuster, 1987; Bansal et al., 1997). A consistent finding has been that these drugs increase the phosphorylation of some cellular proteins. For example, cromoglycate induced the phosphorylation of several intracellular protein substrates including the erythrocyte band 4.1 group protein moesin in rat mast cells (Theoharides et al., 1980; Theoharides et al., 2000). Theoharides et al. (2000) have identified four phosphoproteins with molecular weights of 78, 68, 59, and 42 kDa in mast cells treated with cromoglycate. The authors suggested that the 78 kDa phosphoprotein (later cloned and characterized as cellular cytoskeletal protein moeisin) might be the molecular switch that regulates the degranulation process in mast cells, since the appearance of this protein coincide with the secretory phase stimulated by compound 48/80. The authors speculated that conformational changes of moesin at differential phosphorylation sites regulates the mast cell secretory mechanism, and PKC was identified to be the most likely kinase to be involved in this phosphorylation process (Wang et al., 1999).

PKC has also been associated in the action of cromoglycate in several different models by other investigators (Sagi-Eisenberg, 1985). Some reports have shown that PKC is inhibited by cromoglycate, however discrepancies between the time courses and methodologies obscure clear interpretation of these results.

### GPR 35 Activation

A novel mechanism of cromone action was proposed by two groups (Jenkins et al., 2010; Yang et al., 2010), who suggested that they act through GPR35, a G-protein coupled receptor that modulates signaling via the Gi pathway. This GPCR was previously regarded as an orphan receptor, and its endogenous ligands have been identified as products of tryptophan metabolism, such as kynurenic acid, although fairly high concentrations of these ligands are required to activate the receptor (Wang et al., 2006).

Yang et al. (2010) demonstrated that cromoglycate, nedocromil and zaprinast (another non-cromone anti-allergic) increased inositol phosphate accumulation and calcium mobilization in HEK cells transfected with GPR35. Although there were differences in the specificity exhibited by these drugs when tested on human, mouse and rat GPR35, all three drugs exerted similar potency.

Another study identified a range of ligands including dicoumarol, cromoglycate, and zaprinast in HEK cells transfected with human and rat GPR35, using a β-arrestin-2 interaction assay. Dicoumarol was a partial agonist whilst cromoglycate and zaprinast were full agonist (Jenkins et al., 2010).

GPR35 is present on human mast cells (particularly following treatment with IgE), as well as on eosinophils and basophils. However, its significance to asthma and allergy or to mast cell mediator release is yet to be elucidated, thus it is unclear how these actions of the cromones could be translated into therapeutic effects. Nevertheless, one of the downstream effects of GPR35 activation is activation of PKC so this mechanism would certainly contribute to the observed changes of phosphoprotein abundance in cells.

### The Anx-A1/FPR System

In recent years, our laboratory has proposed a new mechanism to account for cromone action. According to this hypothesis, these drugs activate an endogenous anti-inflammatory loop, the *Anx-A1/FPR* system, which regulates cell activation in many cell types (Yazid et al., 2010b; Sinniah et al., 2016). Interestingly, there are other reports (Oyama et al., 1997; Shishibori et al., 1999) of an association between the annexin family and cromoglycate drugs, whereby these drugs were observed to have an affinity for S100 proteins. It is worth noting that S100 proteins are intracellular binding partners for some members of the annexin family and play a significant role in membrane fusion events (Rintala and Lindahl, 2001).

Anx-A1 is a 37 kDa monomeric protein that is commonly found in many differentiated cells, mainly those of the myeloid lineage (Perretti and D’Acquisto, 2009). It is a member of a superfamily of proteins, which are of ancient evolutionary origin and which are common in most eukaryotic cells (Gerke and Moss, 2002). Structurally, annexins contain a number of homologous core domain repeats attached to an N-terminus of differing lengths that contributes to the various diversity between annexin isoforms (Moss and Morgan, 2004). In mammals, there are 12 annexins and Anx-A1 (numbering corresponds to the first one to be cloned) has four conserved repeats in the core domain. These units has binding motifs for calcium, phosphatidylserine and negatively charged phospholipids (Raynal and Pollard, 1994).

Prior to its cloning, sequencing and characterization (Wallner et al., 1986), Anx-A1 (also known in the older literature as ‘macrocortin’, ‘renocortin’, ‘lipomodulin’, and ‘lipocortin’) was recognized by its characteristic biological activity (Blackwell et al., 1980; Hirata, 1981; Russo-Marie and Duval, 1982; Pepinsky et al., 1986). It was first detected in the conditioned media or perfusate of tissues or cells upon treatment with glucocorticoids, and was found to mirror the action of these drugs in numerous *in vitro* and *in vivo* systems (Pepinsky et al., 1986). The synthesis and release of this protein were hypothesized to account for some of the anti-inflammatory actions of these drugs and this was confirmed by later experiments using the highly purified r-hu-Anx-A1 (Cirino and Cicala, 1993; Perretti et al., 1993; Wu et al., 1995; D’Amico et al., 2000; Gavins et al., 2003; Bandeira-Melo et al., 2005), Anx-A1-deficient transgenic animals (Roviezzo et al., 2002; Croxtall et al., 2003; Hannon et al., 2003), neutralizing antibodies (Croxtall and Flower, 1992; Duncan et al., 1993; Perretti et al., 1996; Teixeira et al., 1998), and anti-sense agents (Croxtall and Flower, 1994; Taylor et al., 1997). Anx-A1 is now known to exert a key ‘anti-inflammatory and pro-resolution’ role in several important host defense responses including acute inflammation (Perretti and D’Acquisto, 2009) and T-cell signaling (D’Acquisto et al., 2008).

The mechanism by which glucocorticoids (and other factors) promote the actions of Anx-A1 has also been investigated in detail (Croxtall et al., 1996, 2000; Parente and Solito, 2004). In many systems including cells of the innate immune system (e.g., macrophages), glucocorticoids have not only been found to promote Anx-A1 synthesis through genomic action, but also to trigger the rapid release of pre-existing pools of Anx-A1 from the cell cytoplasm (Croxtall et al., 2000). The latter mechanism is modulated by PKC, whereby phosphorylation of Anx-A1 at Ser*^27^* and other residues initiates the translocation of Anx-A1 to the plasma membrane and subsequently its release from the cell (Solito et al., 2006). Anx-A1 can then interact with its target cells in an autocrine or paracrine manner by activating receptors of the FPR family, probably ALX/FPR2 in man or, in the mouse, its homolog Fpr2 (Walther et al., 2000; Dufton et al., 2010). Recent literature has shown that FPR2 and FcεR1 co-localize when mast cells are stimulated with ***N-****formyl-methionyl-* leucyl-phenylalanine (fmlp) and IgE antigen, leading to mast cell activation (Xue et al., 2007). This adds to the conundrum on how FPR could be involved in both the activation and inhibition of mast cell degranulation? One unique feature of FPR2 is that it recognizes both pro-inflammatory and anti-inflammatory signals. Liganding of this receptor by Anx-A1 causes it to dimerize, activating a downstream signaling pathway and producing the generally inhibitory effect that Anx-A1 has on cell activation (Cooray et al., 2013). Interestingly, this receptor can also transduce pro-inflammatory signals: in this case, dimerization does not occur and a different signaling pathway is brought into play.

Proteolysis is vital in determining the extent of secreted Anx-A1 action and disturbances of this process may be a trigger in some diseases. In Wegener’s granulomatosis (for example), extreme PMN activation could be trailed down to abnormal Anx-A1 cleavage by the PR3 protease (Pederzoli-Ribeil et al., 2010).

Mast cells, one of the principal targets of cromoglycate action contain abundant Anx-A1 and respond rapidly to glucocorticoid treatment with an increase in Anx-A1 mRNA (Oliani et al., 2000). These cells also express the Anx-A1 receptor, ALX/FPR2. The sub-cutaneous injection of the mast cell secretagogue compound 48/80 into the flank of mice produces a greater wheal in Anx-A1 null mice compared to its wild type counterpart (Yazid et al., 2010a). In addition, mast cells in Anx-A1 ‘knock-out’ mice are more susceptible to degranulation, hence release more PGD_2_ and histamine than wild type cells in response to degranulating stimulants (Damazo et al., 2005). The acute inhibitory action of cromones as well as glucocorticoids on mast cell mediator release is blocked in the presence of specific anti-Anx-A1 neutralizing antibodies (Sinniah et al., 2016). All these observations suggest that Anx-A1 exerts a ‘homeostatic’ inhibitory influence on mast cell reactivity and is important in the mode of action of the cromones.

To further support this notion, a complementary study in which administration of the biomimetic Anx-A1 N-terminal peptide Ac 2-26 inhibited IgE-induced histamine release in the pleural cavity (Bandeira-Melo et al., 2005) strongly suggests that Anx-A1 is also an endogenous regulator of mast cell degranulation in an experimental model of rat allergic pleuritis.

### Action of Cromones on the PP2A Phosphatase

In view of the fact that the cromoglycate-like drugs act as mast cell stabilizers, and that Anx-A1 is clearly implicated in mast cell biology, an obvious hypothesis was that the cromoglycate-like drugs could release Anx-A1, which then mediated the pharmacological actions of these drugs. A simple model for studying the biological actions of Anx-A1 release under closely defined conditions was utilized to investigate this possibility (Yazid et al., 2009).

U937 cells were used as these cells are responsive to glucocorticoids when differentiated, contains abundant Anx-A1 and produces a convenient biochemical readout of activation. This study concluded that thromboxane (Tx) B_2_ production was suppressed when glucocorticoids such as dexamethasone initiate the phosphorylation and release of Anx-A1, that the phosphorylation of Anx-A1 precedes export and that PKC was the kinase most likely responsible for this action. Occupancy of the glucocorticoid receptor prompts PKC activation. Although the mechanism is not well elucidated, some observations have implicated the involvement of PIP3 kinase in this activation (Solito et al., 2003).

**FIGURE 1:**
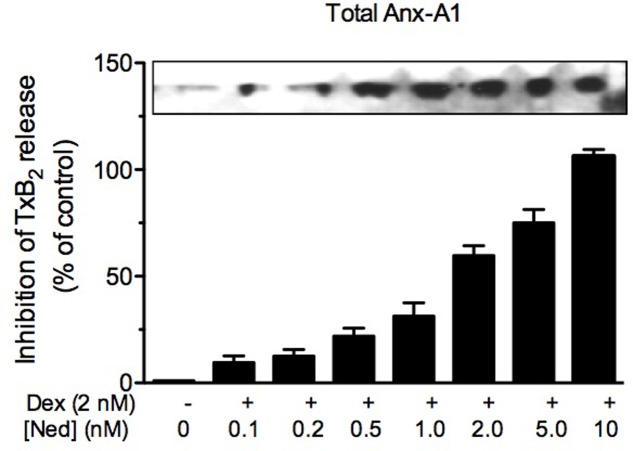
Nedocromil potentiates dexamethasone inhibition of TxB_2_ release from U937 cells. The increased internalization of Anx-A1 (as assessed by western blotting) and concomitant inhibition of TxB_2_ release (as assessed by ELISA assay) produced by escalating concentrations of nedocromil (0.2–10 nM) in the presence of a fixed concentration (2 nM) of dexamethasone. Figure reproduced with the permission from the rights holder, Elsevier (Yazid et al., 2009).

Yazid et al. (2009) had speculated that some interaction occurred between the acute effect of dexamethasone and the action of cromoglycate, although the reason for this interaction was unclear at that time. Analysis of Anx-A1 in U937 cells revealed that whilst dexamethasone produced a small increase in phosphorylated and externalized Anx-A1, the addition of cromoglycate alone was without effect. A study of the dynamics of the glucocorticoid-induced phosphorylation of Anx-A1 at the Ser^27^ residue showed that the effect seen was very rapid with a maximum activation of PKC and Anx-A1 phosphorylation occurring within 5 min. Whilst the cromones alone had little effect on Anx-A1 phosphorylation, they greatly potentiated the effect of the glucocorticoids (see **Figure 1**). This was evident by the redistribution of GFP-tagged Anx-A1 within the U937 cells, extracellular accumulation of the protein and, dramatically, by the observed inhibition of TxB_2_ generation. For example, the inhibition of TxB_2_ release in U937 cells treated with 2 nM of dexamethasone alone was only <20%, but this inhibition was increased to almost 100% in the presence of 20 nM nedocromil at 5 min time point. Cromolycate also has similar glucocorticoid potentiating ability.

The regulation of PKC activity may be accomplished through several potential mechanisms. The duration of action of PKC is limited by phosphatases such as PP2A. PKCα (and some other isoforms) interact with PP2A following activation in many cell types, including macrophage (see review Sim and Scott, 1999). PKC activation is followed by its translocation to the plasma membrane (see Refs. Ishizuka et al., 1995; Park et al., 2001; Qiu et al., 2003; Plotkin et al., 2007) where its catalytic activity is terminated by the phosphatase, which limits the duration of its biological actions (Sagi-Eisenberg, 1985; Ricciarelli and Azzi, 1998; Boudreau et al., 2002; Faulkner et al., 2003; Zhang et al., 2007; Lee et al., 2008). Another possibility is modulation of diacylglycerol (DAG) activity. DAG is the endogenous activator of PKC, but is rapidly destroyed by DAG kinases which therefore also terminate PKC activation. It follows that inhibitors of either of these two processes should greatly potentiate the glucocorticoid-induced increase in Anx-A1 phosphorylation and release from cells. One possibility therefore was that the cromones were in fact inhibitors of PP2A.

Previous experimental observations had revealed that GC receptor ligation, perhaps acting through phosphatidylinositol 3-kinase (Solito et al., 2003), causes PKC activation and membrane translocation and that this activity was limited by dephosphorylation, through the Ser/Thr PP2A phosphatase (Hansra et al., 1996). Further support for a mechanism of this type in the U937 cell system was obtained in an experiment with the PP2A inhibitor okadaic acid (OA). Treatment of these cells with OA greatly potentiated the effect of dexamethasone on Anx-A1 externalization and eicosanoid synthesis (Yazid et al., 2009).

This hypothesis was further tested by assessing the ability of nedocromil and cromoglycate to inhibit the endogenous phosphatase activity found in the membrane fraction of U937 cells following treatment with dexamethasone. These drugs strongly inhibited this phosphatase activity and similar findings were reported when highly purified recombinant PP2A was used as a target enzyme (Yazid et al., 2009).

PP2A is a heterotrimeric enzyme comprising one each of two variant catalytic and structural sub-units together with one (of a family of about twenty) modulatory/targeting sub-unit, which determines the specificity of the assembled enzyme complex. C-terminal carboxymethylation of the catalytic sub-unit at Leu^309^ activates PP2A probably by facilitating the formation of trimeric complexes (Lee et al., 2007; Yoo et al., 2007; Ortega-Gutiérrez et al., 2008). The enzyme may be phosphorylated on Tyr^307^ by either receptor or other tyrosine kinase action (Chen et al., 1992) which may be the way in which the phosphatase activity itself is terminated at the membrane. However, the importance of these post-translational modifications to enzyme activity *in vitro* and *in vivo* is not yet entirely clear (Sim and Scott, 1999).

It is likely from the available experimental evidence that the cromones interact with the catalytic site of the trimeric PP2A complex. This contains Mn^2+^ which might possibly be a target for the drugs which could conceivably interact with this metal being carboxylic acids (unpublished data). Interestingly, previous authors had already investigated the possibility that cromones inhibited PP2A, but were unable to detect an effect in their system (Wang et al., 1999). However, some groups had noted a link between the action of these drugs *in vivo* and inhibition of alkaline phosphatase in a rat passive cutaneous anaphylaxis model (Schwender, 1981; Schwender et al., 1982).

**FIGURE 2:**
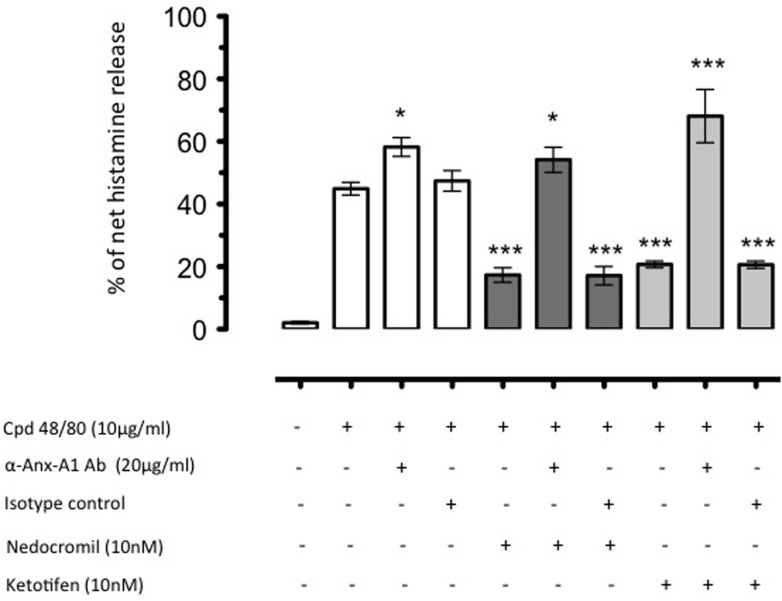
The inhibition by nedocromil and ketotifen of mediator release from Cord blood derived mast cells (CBDMCs) stimulated with compound 48/80 is dependent upon Anx-A1. CBDMCs were plated at a density of 2 × 10^5^ cells per well and the stipulated groups were treated with 20 μg/ml Anx-A1 neutralizing antibody or an irrelevant isotype control. Subsequently, the cells were pre-treated with either nedocromil (10 nM) or ketotifen (10 nM) for 5 min followed by compound 48/80 (10 μg/ml) stimulation for 10 min. To assess the effects of Anx-A1 removal, the cells were incubated with the Anx-A1 neutralizing antibody (or an irrelevant isotype control) only. Nedocromil and ketotifen produced consistent inhibition of histamine, but not control isotype matched non-neutralizing mAb. Data are expressed as mean ± SEM from *n* = 3 experiment and were analysed using one-way analysis of variance (ANOVA), followed by a Bonferroni *post hoc* test, ^∗^*p* < 0.05, ^∗∗∗^*p* < 0.001 vs unstimulated). Figure reproduced with the permission from the rights holder, Elsevier (Sinniah et al., 2016).

Increased Anx-A1 release reflected the inhibitory actions of combined nedocromil and dexamethasone treatment (Sinniah et al., 2016). An additional interesting observation from this study was that depletion of Anx-A1 *in vitro* completely reversed the inhibitory actions of these drugs (see **Figure 2**). This mechanism was also observed to operate *in vivo.* Using Anx-A1 null mice, the ability of nedocromil to inhibit leukocyte migration in a model of peritoneal inflammation in mice was lost whilst they were fully active in the wild type (Yazid et al., 2010b).

### Other Drugs Which May Operate Using the Anx-A1/FPR Pathway

Several other drugs, including some non-cromone anti-allergic/anti-histamines (including azelastine, pemirolast, and olopatidine) exhibit cross-tachyphylaxis, or share a similar pharmacology, with cromones (Cook et al., 2002).

One such drug is the second-generation H_1_ antagonist ketotifen. When tested, ketotifen was found to have the same effect on the Anx-A1 system as nedocromil and cromoglycate suggesting a commonality of mechanisms of action (Sinniah et al., 2016). An important speculation is that all H_1_ antagonists with this additional action may have a secondary pharmacology as PP2A inhibitors and, if so, this could be a beneficial therapeutic screen to evaluate this property.

### The Future of Cromone Pharmacology

The demonstration by our group that the cromones potentiate the acute effect of glucocorticoids on Anx-A1 release could have some clinical implications as Anx-A1 has been implicated in the regulation of bronchial hyper-reactivity and asthma, at least in animals (Ng et al., 2011). It begs the question of whether using a mixture of the two drugs would augment the response to glucocorticoids perhaps permitting a reduction in the dose required to achieve a therapeutic effect. An understanding of the mechanism of action of these drugs will enable – for the first time – the creation of a simple biochemical screen or readout for testing future cromone or other derivatives with presumed anti-allergic properties.

Of course, Anx-A1 is not the sole substrate for PKC, so other PKC-dependent phosphorylation events might be potentiated in the presence of the cromones. We have already mentioned an early observation that increases in the phosphorylation of moesin and several other proteins have been observed in the presence of these drugs. What other targets might there be in the cell? One significant target may be the glucocorticoid receptor. Whilst the acute increase in Anx-A1 secretion we have observed is due entirely to an increased secretion of the protein, we are aware that the glucocorticoid receptor itself is phosphorylated upon liganding, possibly by PKC, and that PP2A (which is associated with the receptor complex) plays a key role in terminating its activation after an appropriate time period (Kobayashi et al., 2017). One might predict changes in glucocorticoid receptor behavior in the presence of cromones although how this would play out in terms of their therapeutic action will have to be investigated.

**FIGURE 3:**
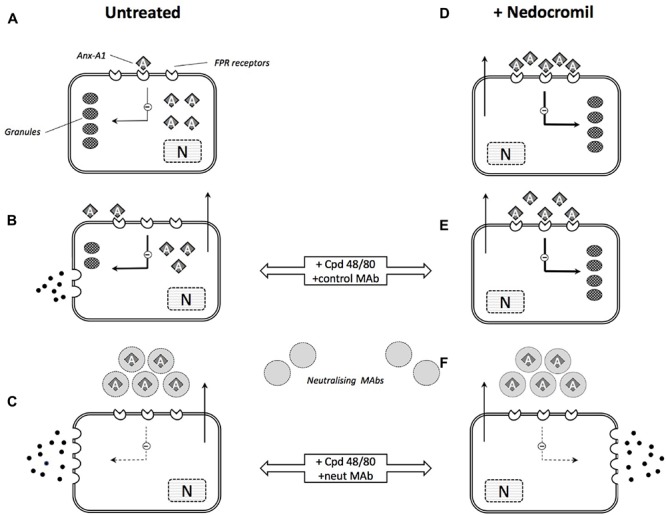
Schematic illustration of the role of Anx-A1 in mast cell degranulation and cromone action. **(A)** In the untreated, ‘resting’ mast cell, there is an intracellular pool of Anx-A1, a small proportion of which is externalized exerting a low-level tonic inhibitory influence on cell activation. **(B)** During stimulation by (e.g., Cpd 48/80) granule contents are released but Anx-A1 is also phosphorylated and released as a result of PKC activation. This provides some feedback control over the extent of degranulation. **(C)** In the presence of neutralizing anti-Anx-A1 mAbs, this feedback control is lost and degranulation is more extensive. **(D)** In mast cells pre-treated with nedocromil or cromoglycate, Anx-A1 is already externalized and fully engaged with inhibitory FPR receptors. This suppresses degranulation in response to degranulating stimuli **(E)** by inhibiting the activation response and ‘stabilizing’ the mast cell. **(F)** In the presence of the neutralizing mAb, however, this inhibitory influence is removed and extensive degranulation occurs.

The possible mechanism of action by cromone in mast cells is summarized in **Figure 3**.

## Conclusion

To conclude, we have reviewed evidence supporting the hypothesis that Anx-A1 is indeed a crucial endogenous regulator of mast cell function, which might reciprocally inhibit mast cell activation. Anx-A1 is secreted in increased amounts parallel with mediators during mast cell activation and thus, acts to control the extent of mast cell degranulation and activation response.

We also described a different, GPR35-dependent mechanism, which could also transduce some of the effects of the cromones. In trying to reconcile these two ideas, it is worth noting that the effects on the Anx-A1 system that was reported here was very rapid (within 5 min) and that we have not looked at other actions of the cromones that may require a longer latent period, which could be mediated by an alternative GPR35 mechanisms.

## Author Contributions

All the authors contributed to the writing of the manuscript. AS prepared the final version and prepared the paper for publication.

## Conflict of Interest Statement

The authors declare that the research was conducted in the absence of any commercial or financial relationships that could be construed as a potential conflict of interest.
